# Successful management of refractory pleural effusion due to systemic immunoglobulin light chain amyloidosis by vincristine adriamycin dexamethasone chemotherapy: a case report

**DOI:** 10.1186/1752-1947-4-322

**Published:** 2010-10-18

**Authors:** Toshikazu Araoka, Hiroya Takeoka, Keisuke Nishioka, Masaki Ikeda, Makiko Kondo, Azusa Hoshina, Seiji Kishi, Makoto Araki, Rokuro Mimura, Taichi Murakami, Akira Mima, Kojiro Nagai, Hideharu Abe, Toshio Doi

**Affiliations:** 1 Deapartment of Nephrology, Graduate School of Medicine, Institute of Health-Bio-Science, University of Tokushima, Tokushima, Japan; 2 Division of Nephrology, Hyogo Prefectural Amagasaki Hospital, Hyogo, Japan; 3 Division of Pathology, Hyogo Prefectural Amagasaki Hospital, Hyogo, Japan; 4 Department of Nephrology, Graduate School of Medicine, University of Kyoto, Kyoto, Japan

## Abstract

**Introduction:**

Refractory pleural effusion in systemic immunoglobulin light chain amyloidosis without cardiac decompensation is rarely reported and has a poor prognosis in general (a median survival of 1.6 months). Moreover, the optimum treatment for this condition is still undecided. This is the first report on the successful use of vincristine, adriamycin and dexamethasone chemotherapy for refractory pleural effusion due to systemic immunoglobulin light chain amyloidosis without cardiac decompensation.

**Case presentation:**

We report the case of a 68-year old Japanese male with systemic immunoglobulin light chain amyloidosis presenting with bilateral pleural effusion (more severe on the right side) in the absence of cardiac decompensation that was refractory to diuretic therapy. The patient was admitted for fatigue, exertional dyspnea, and bilateral lower extremity edema. He had been receiving intermittent melphalan and prednisone chemotherapy for seven years. One month before admission, his dyspnea had got worse, and his chest radiograph showed bilateral pleural effusion; the pleural effusion was ascertained to be a transudate. The conventionally used therapeutic measures, including diuretics and thoracocentesis, failed to control pleural effusion. Administration of vincristine, adriamycin, and dexamethasone chemotherapy led to successful resolution of the effusion.

**Conclusion:**

Treatment with vincristine, adriamycin, and dexamethasone chemotherapy was effective for the refractory pleural effusion in systemic immunoglobulin light chain amyloidosis without cardiac decompensation and appears to be associated with improvement in our patient's prognosis.

## Introduction

Systemic immunoglobulin light chain (AL) amyloidosis is a rare disorder characterized by the extracellular deposition of amyloid fibrils resulting from the formation of insoluble aggregates of β-pleated sheets, which are derived from monoclonal light chains. It may lead to progressive multiple-organ failure and death.

Recent studies have suggested that the prognosis of systemic AL amyloidosis without pleural effusion is improved by novel approaches, including vincristine, adriamycin, and dexamethasone (VAD) chemotherapy and high-dose myeloablative chemotherapy with autologous stem cell transplantation support. In particular, the median duration of patient survival with VAD chemotherapy (64 to 65 months) is higher than that with intermittent melphalan and prednisone (MP) chemotherapy [1].

However, little is known concerning whether the refractory pleural effusion in systemic AL amyloidosis without cardiac decompensation is associated with a poor prognosis in general (a median survival of 1.6 months) because this complication is rarely reported (6%) [2]. Hence, new therapies and the mechanisms of systemic AL amyloidosis with refractory pleural effusion needs to be discussed further.

Although the effectiveness of the above-mentioned therapies for pleural effusion in systemic AL amyloidosis has been evaluated by some previous studies [2-6], the most effective therapy remains unidentified. Pleurodesis is often required because pleural effusion is refractory to most therapies. Although pleurodesis temporarily alleviates the symptoms, its effectiveness for improving the prognosis of systemic AL amyloidosis remains to be clarified.

We used chemotherapy for the first time to successfully manage refractory pleural effusion due to systemic AL amyloidosis without cardiac decompensation. VAD chemotherapy may improve the prognosis of this complication.

## Case presentation

A 68-year-old Japanese male with systemic AL amyloidosis was admitted with fatigue, exertional dyspnea, and bilateral lower-extremity edema. He had been diagnosed with systemic AL amyloidosis in 1999, on the basis of the results of histopathological examination of the biopsied tissues of his kidney and bone marrow. After diagnosis, he was successfully managed with 21 courses of intermittent MP chemotherapy for seven years. Two months before admission, he developed exertional dyspnea. One month before admission, his dyspnea had become worse, and his chest radiograph showed bilateral pleural effusion, with the effusion on the right side being more severe. Thoracocentesis was performed with removal of one litre of yellow serous fluid from the right hemithorax. Pleural fluid analysis revealed that his nucleated cell count was 2600/mm^3 ^(24% neutrophils, 71% lymphocytes); total protein concentration, 0.4 g/dl (a pleural fluid to serum ratio, 0:11); lactate dehydrogenase level, 26 IU/l (two-thirds normal upper limit for serum, 154 IU/l and a pleural fluid to serum ratio, 0:12); total cholesterol content, 11 mg/dl; and glucose level, 118 mg/dl. These findings were consistent with transudative pleural effusion. The pleural fluid cytology was negative for malignancy. The pleural fluid was cultured for bacteria (aerobic and anaerobic), fungi, and mycobacteria, and the results were negative.

Since the pleural effusion was refractory to aggressive administration of diuretics, thoracentesis with removal of one litre of serous fluid was repeated once every week. Although his symptoms were alleviated, the patient's pleural effusion gradually increased and the pleural fluid returned to the pre-drainage level after one week. The composition of the pleural fluid remained the same at all instances of drainage.

He was admitted to our hospital for treatment of refractory pleural effusion. On admission, he appeared to be comfortable while resting. He was 158.7 cm tall and weighed 65.2 kg. His body temperature was 36.5°C; blood pressure was 124 over 69 mm Hg; and his pulse rate, regular at 84 beats per minute. Diminished breath sounds in the right lower lung, slight inspiratory coarse crackles in the left basal lung, and lower extremity edema were observed. The results of the other clinical examinations were normal. Laboratory tests revealed that his blood urea nitrogen (31 mg/dl) and serum-creatinine (1.3 mg/dl) levels were elevated; creatinine clearance rate was low (39 ml/min/1.73 m^2^); 24-hour urine protein was 3.5 g per day; and total protein content (4.6 g/dl) and serum-albumin levels (1.6 g/dl) were low. These findings were indicative of renal insufficiency and nephritic syndrome. However, his renal function and his serum-albumin levels remained the same as those observed one year before. The serum protein electrophoresis was negative for a monoclonal spike. Bence-Jones proteins were not detected in the urine. All his immunoglobulin levels were normal. His hemoglobin level was 12.4 g/dl, platelet count 12 × 10^4^/mm^3^, and white blood cell count, 5900/mm^3^, with a normal differential count. His levels of troponin T (below 0.01 mg/dl) and the C-reactive protein (0.2 mg/dl) were also normal. No other abnormalities were detected in any other laboratory tests. Our patient's echocardiogram showed symmetrical thickening of the left ventricular (LV) wall with slightly high echoic lesions (interventricular septum wall thickness, 14 mm; posterior LV wall thickness, 15 mm at systolic phase) and almost normal cardiac function (fractional shortening, 40%; LV ejection fraction, 0.71). These results were suggestive of infiltrative cardiomyopathy, and not acute heart failure caused by cardiac compensation.

**Figure 1:**
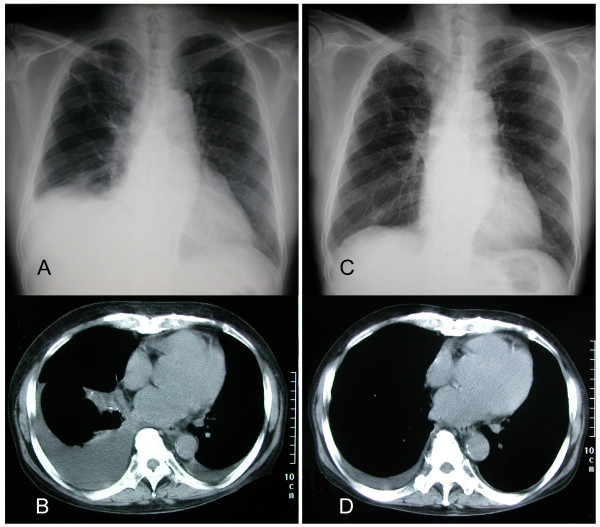
**Pleural effusion was significantly alleviated by vincristine, adriamycin and dexamethasone chemotherapy**. Chest radiograph **(A) **and computed tomography imaging **(B) **showing pleural effusion at admission. Chest radiograph **(C) **and computed tomography imaging **(D) **showing significant improvement 30 days after VAD.

At admission, his chest radiograph image showed a moderate effusion on the right side and slight effusion in the left. The computed tomography imaging of his chest revealed moderate pleural effusion in the right lung and atelectasis of the right lower robe (Figure 1A, B).

Thoracentesis was performed with removal of one litre of serous fluid from the right hemithorax on the second hospital day. The level and composition of the effusion were the same as those seen before. VAD chemotherapy was administered on the third day. He did not develop serious bone marrow suppression or complications. A week after the administration of VAD chemotherapy, his chest radiograph revealed significant improvement. Moreover, he did not have dyspnea or general fatigue. However, the edema in his lower extremities showed no improvement, and no obvious change was seen in cardiac indices; pleural effusion did not recur until he was discharged from the hospital (Figure 1C, D). The results of the clinical test did not change (creatinine clearance rate, 35 ml/minute/1.73 m^2^; daily proteinuria, 3.4 g; serum-albumin, 1.6 g/dl). He was discharged from our hospital on his 31^st ^hospital day.

Since then, he has continued receiving intermittent MP chemotherapy. Pleural effusion did not recur for six months after he became ambulatory. However, he had a recurrence of right pleural effusion and was managed with VAD chemotherapy. Although his right pleural effusion did not increase while treating with VAD chemotherapy, the recurrence occurred immediately after VAD chemotherapy had finished. Hence, in 2006, he underwent chemical pleurodesis for recurrent right pleural effusion. After pleurodesis, his pleural effusion has not increased after he received intermittent MP chemotherapy in 2010.

## Discussion

Pleural effusion in systemic AL amyloidosis is generally considered to be caused by heart failure, nephritic syndrome, and renal insufficiency. The poor prognosis of pleural effusion is attributed to cardiac amyloidosis. However, earlier studies have reported that in the absence of cardiac decompensation due to cardiac amyloidosis, the prognosis for untreated patients with pleural effusion is shorter than that for patients without pleural effusion (1.6 months vs. 6 months: a median survival) [2]. Moreover, pleural effusion in the patient in our report was completely cured by one cycle of VAD chemotherapy with no change in serum-albumin levels and cardiac and renal function. These findings imply that refractory pleural effusion associated with systemic AL amyloidosis should be considered a manifestation of the disease just like cardiac and renal amyloidosis and not as one of the symptoms of cardiac decompensation and renal disorder.

Earlier reports stated that refractory pleural effusion in systemic AL amyloidosis tended to be treated by pleurodesis. Although pleurodesis represents the gold standard for the treatment of massive recurrent pleural effusion, this procedure is invasive and may be associated with risks of infection and pneumothorax [2]. Hence, to manage the refractory pleural effusion, we administered VAD chemotherapy as an initiation therapy before pleurodesis.

This is the first report on the successful use of VAD chemotherapy for refractory pleural effusion in systemic AL amyloidosis without cardiac decompensation. This therapy was not invasive and reduced pleural effusion within a short time. Hence, VAD chemotherapy may become an effective treatment for refractory pleural effusion resulting from systemic AL amyloidosis. However, whether VAD chemotherapy can be used as the initial therapy before pleurodesis remains to be clarified. Doxorubicin, vincristine, and dexamethasone, which are included in the VAD regimen, can cause cardiac toxicity, neuropathy, and infection, respectively. Therefore, VAD chemotherapy should be administered after careful consideration.

Infiltrative cardiomyopathy revealed in an echocardiogram may be caused by the amyloid deposition, which has the potential to alter regional cardiac mechanics, resulting in LV dyssynchrony and cardiac decompensation [7]. Although the mechanisms by which VAD ameliorate the infiltrative cardiomyopathy are still unclear, a recent report suggests that the reduction of amyloid depositions resulting from decreased precursor protein may be the mechanism by which VAD exert their therapeutic effects [8]. The cardiac function of this patient remained unchanged after administration of VAD chemotherapy; however, a similar pathogenic mechanism may be involved in the development of refractory pleural effusion without cardiac decompensation. Previous reports on pathological findings of tissue samples obtained by needle biopsies have indicated that amyloid depositions in the parietal pleura play an important role in the pathogenesis of pleural effusion by inhibiting the absorption of pleural fluid by interfering with the lymphatic drainage [2,3,6,9]. Hence, amyloid depositions in the parietal pleura may be directly reduced by VAD chemotherapy. However, we thought that pleural effusion was not a direct effect of amyloid protein accumulation in the lymph duct because one cycle of VAD chemotherapy alleviated the pleural effusion in our patient within a week. We hypothesized that the indirect effect of amyloid deposition was more important than its direct effect on the pathogenesis of pleural effusion.

Several reports have suggested that pleural effusion may be caused by increased permeability of the pleural capillaries [9]. Moreover, it has been reported that vascular endothelial growth factor (VEGF) expression increased in systemic AL amyloidosis [10] and that bevacizumab (a monoclonal antibody against VEGF) was effective in the management of refractory pleural effusion [4,5]. Therefore, we thought that VAD chemotherapy may be as effective as bevacizumab in alleviating pleural effusion by inhibiting VEGF expression. The mechanisms of their action need to be elucidated further.

We report that thalidomide [11], lenalidomide (an analog of thalidomide) [12] and bortezomib (a proteosome inhibitor) [13] may have potentially important roles for treating refractory pleural effusion accompanying systemic AL amyloidosis because these drugs have been reported to inhibit the expression of VEGF [14-16]. It has been observed that these drugs pose a lower risk of infection than conventional chemotherapy. Hence, these drugs may represent important alternative therapeutic agents for alleviating refractory pleural effusion due to systemic AL amyloidosis. In addition, a combination of these drugs and conventional chemotherapy may improve the prognosis of this disease.

However, further investigations are needed to elucidate the relationship between VEGF and refractory pleural effusion associated with systemic AL amyloidosis because the complete reduction of pleural effusion was observed in only two of the five patients reported to be treated with bevacizumab [4,5].

## Conclusion

Management of patients with systemic AL amyloidosis who present with refractory pleural effusion is extremely difficult and is associated with a poor prognosis. To the best of our knowledge, this is the first report on the administration of VAD chemotherapy for refractory pleural effusion in systemic AL amyloidosis without cardiac decompensation. This treatment may be effective and afford a high quality of life for the patient.

In general, VAD chemotherapy is known to improve the prognosis of systemic AL amyloidosis, and the Guideline Working Group of United Kingdom, Myeloma Forum, recommends VAD chemotherapy as the first-line therapy for patients aged under 70 years [17]. Therefore, we suggest that VAD chemotherapy is clinically useful for regulating systemic AL amyloidosis-associated refractory pleural effusion.

## Abbreviations

LV: left ventricular; MP: melphalan and prednisone; VAD: vincristine, adriamycin, and dexamethasone; VEGF: vascular endothelial growth factor

## Competing interests

The authors declare that they have no competing interests.

## Consent

Written informed consent was obtained from the patient for publication of this case report and any accompanying images. A copy of the written consent is available for review by the Editor-in-Chief of this journal.

## Authors' contributions

TA, HT, HA and TD were the major contributors to writing the manuscript. TM, AM and KN contributed to the discussion of the revised version of the manuscript. KN, MI, MK and AH structured the management plan and did follow-up on the patient. SK and MA supervised the chemotherapy. RM performed the histological examination of the biopsied tissues of the patient's kidney and bone marrow. All authors read and approved the final manuscript.
